# The Role of Human Leukocyte Antigen (HLA) Allele Patterns in the Incidence of End-Stage Renal Disease and HLA Antibody Production: Protocol for a Case-Control Study

**DOI:** 10.2196/107508

**Published:** 2026-09-30

**Authors:** Ira Puspitawati, Nyoman Kertia, Teguh Triyono

**Affiliations:** 1 Doctoral Program, Faculty of Medicine, Public Health and Nursing, Universitas Gadjah Mada, Yogyakarta, Indonesia; 2 Clinical Pathology and Laboratory Medicine Department, Faculty of Medicine, Public Health and Nursing, Universitas Gadjah Mada/Dr Sardjito Hospital, Jalan Kesehatan, Sekip Utara, Yogyakarta, 55281, Indonesia, 62 8112655990; 3 Internal Medicine Department, Faculty of Medicine, Public Health and Nursing, Universitas Gadjah Mada/Dr Sardjito Hospital, Yogyakarta, Indonesia

**Keywords:** end-stage renal disease, human leukocyte antigen, human leukocyte antigen antibody, risk factor, HLA polymorphism, HLA

## Abstract

**Background:**

The incidence of end-stage renal disease (ESRD) continues to increase globally, including in Indonesia, representing a significant public health burden. Genetic factors, particularly variations in human leukocyte antigen (HLA) alleles, have been suggested to contribute to ESRD susceptibility, either indirectly through their association with underlying immune-mediated diseases or directly through their involvement in immune regulation and renal injury pathways. Studies have shown varying results due to HLA polymorphisms, ethnicity, and environmental influences. HLA polymorphisms are suspected of influencing HLA antibody production, which in turn impacts kidney transplant outcomes. Although ethnicity is one of the factors that determine HLA allele variation in the population, research on HLA alleles has not been widely conducted in Indonesia.

**Objective:**

This study aims to analyze the role HLA alleles play in the occurrence of ESRD and their association with HLA antibody production.

**Methods:**

This observational case-control study incorporates both retrospective and prospective data collection at Dr Sardjito General Hospital, Yogyakarta. Retrospective HLA typing and HLA antibody data were obtained from the medical records of patients with ESRD and healthy controls between January 2020 and May 2026. Additional participants in both groups are being prospectively recruited between June and December 2026 according to predefined eligibility criteria. The case group will comprise participants aged 18 years or older with ESRD, complete HLA typing data for 8 loci, and available HLA antibody results, excluding those who have undergone kidney transplantation. The control group will comprise individuals aged 18 years or older with normal kidney function. HLA allele typing will be performed using a molecular method, namely, polymerase chain reaction sequence–specific oligonucleotide probes. HLA antibody testing will be performed using a solid-phase assay-based technology, specifically a Luminex single-antigen bead assay (DiaSorin), and both the HLA allele typing and HLA antibody testing will be analyzed using a LABScan3D analyzer (One Lambda Inc). The HLA antibody results will be subsequently categorized into positive and negative calculated panel reactive antibody (cPRA) statuses. Statistical analysis using odds ratio (OR) calculation will be performed to determine the association between specific HLA alleles and the occurrence of ESRD as well as HLA antibody production, with a 95% CI.

**Results:**

Participant recruitment and data collection began after approval of ethical considerations and are anticipated to be completed by December 2026. Dissemination of the study findings is planned for mid-2027.

**Conclusions:**

This study will evaluate the role of HLA class I and class II allele patterns in the development of ESRD and anti-HLA antibody production, as well as the influence of demographic characteristics, sensitization-related factors (including blood transfusion, pregnancy, and autoimmune disease), and clinical comorbidities on HLA antibody production and susceptibility to ESRD.

## Introduction

### Background

According to the Indonesian Basic Health Research Survey (Riskesdas) conducted by the Ministry of Health in 2018, 739,208 individuals, representing 3.8 per 1000 population in Indonesia, have chronic kidney disease (CKD). This is a significant increase compared with the 2013 Riskesdas survey, which reported a prevalence of 2 per 1000 population. This trend was accompanied by an increasing number of cases progressing to end-stage renal disease (ESRD) [1]. Data from the Indonesian Renal Registry demonstrate that the incidence of new ESRD cases increased from 9,649 in 2010 to 30,831 in 2017. Similarly, the prevalence of ESRD rose from 11,484 cases in 2010 to 77,829 cases in 2017 [2]. Furthermore, data from the Indonesian Society of Nephrology (PERNEFRI) showed that by 2020, the cumulative incidence of patients undergoing dialysis had reached 61,786, while the cumulative prevalence had increased to 130,931 individuals [3].

The Kidney Disease: Improving Global Outcomes guidelines define CKD as a decline in kidney function (estimated glomerular filtration rate [eGFR] <60 mL/min/1.73 m²) and the presence of proteinuria (albumin-to-creatinine ratio >30 mg/g), accompanied by signs of structural kidney abnormalities persisting for at least 3 months. ESRD corresponds to stage 5 CKD and is characterized by an eGFR of less than 15 mL/min/1.73 m² of body surface area [4].

The etiology of ESRD is complex and multifactorial, encompassing both genetic and acquired conditions. Mendelian genetic disorders are estimated to account for more than 10% of ESRD cases, while an additional polygenic contribution to ESRD susceptibility is increasingly recognized but remains incompletely characterized. Genetic factors may also contribute to a proportion of ESRD cases currently classified as having an unknown etiology, which has been estimated at approximately 11%. In contrast, the majority of ESRD cases are attributed to acquired or multifactorial conditions, including diabetes mellitus, hypertension, glomerular diseases, infections, drug-induced nephropathy, and other systemic or metabolic disorders. However, the distinction between genetic and nongenetic etiologies is not always definitive because the underlying cause of ESRD may be difficult to establish clinically, and disease progression often results from interactions between genetic susceptibility and environmental or acquired factors. These observations highlight the importance of investigating genetic determinants that may contribute to ESRD susceptibility, including variation within the human leukocyte antigen (HLA) system [5].

Among the genetic determinants, HLA genes play a particularly important role. HLA molecules are encoded by genes located on chromosome 6p21.3 and are responsible for binding and presenting antigens to T lymphocytes, thereby initiating cellular immune responses. HLA molecules also play a critical role in allograft rejection [6]. Two major classes of HLA molecules have been identified. HLA class I molecules are expressed on the surface of nearly all nucleated cells. Among class I loci, HLA-B is the most polymorphic and has been shown to be the most significant, followed by HLA-A and HLA-C. HLA class II molecules are expressed primarily on immunocompetent cells, including B lymphocytes, macrophages, endothelial cells, and activated T lymphocytes [7]. The association between HLA polymorphisms and ESRD may be explained by their relationship with the etiology and progression of kidney diseases [8,9].

In the Indonesian population, diabetes mellitus and hypertension are the leading etiologies of CKD, with reported 1-year and 5-year survival rates of 83% and 51.9%, respectively [2]. Although the contribution of genetic susceptibility remains incompletely characterized, previous research has investigated the potential role of HLA polymorphisms in ESRD among Indonesian patients undergoing transplantation. Susianti et al [10] reported substantial heterogeneity in both HLA class I and HLA class II antigens and identified several HLA polymorphisms associated with ESRD in the Indonesian population. HLA-A*24 and HLA-B*35 were associated with protection against ESRD, whereas HLA-B*13-DRB1*15 was the most frequent 2-locus haplotype associated with susceptibility, and HLA-A*24-B*13-DRB1*15 was identified as the most frequent 3-locus haplotype associated with ESRD susceptibility. These findings suggest that HLA genetic variation may contribute to individual susceptibility to ESRD in the Indonesian population. However, the observed associations require further investigation and validation in larger and more diverse Indonesian populations, particularly given the substantial genetic and ethnic heterogeneity of the Indonesian archipelago. Therefore, comprehensive characterization of HLA polymorphisms across multiple HLA loci may provide further insight into the genetic contribution to ESRD in Indonesia.

### This Study

Several studies have reported associations between specific HLA alleles and the development of ESRD. The HLA alleles previously associated with ESRD have not been consistent across populations, suggesting that HLA-ESRD associations may be population-specific rather than attributable to universally prevalent risk alleles. For example, HLA-DR3 and HLA-DR11 were associated with increased ESRD risk in Taiwan, whereas HLA-DR8 was reported to be protective, while different HLA-A, HLA-B, and HLA-DRB1 alleles were associated with ESRD in Cantonese, Saudi Arabian, Hunan Chinese, and Romanian populations [11-16]. A recent meta-analysis of 26 case-control studies likewise found no consistently significant association for several previously reported HLA alleles, further supporting substantial heterogeneity among populations [17].

A study conducted by Dai et al [12] in Taiwan identified HLA-DR3 (OR 1.90, 95% CI 1.10‐3.32; *P*=.02) and HLA-DR11 (95% CI 1.13‐3.76; *P*=.02) as risk factors for ESRD, whereas HLA-DR8 was found to exert a protective effect (OR 0.47, 95% CI 0.24‐0.92; *P*=.03). In contrast, HLA-A and HLA-B were not associated with ESRD susceptibility. Cao et al [13] reported that 5 HLA alleles (HLA-A*24, HLA-B*55, HLA-B*54, HLA-B*40(60), and HLA-DRB1*04) as well as 1 haplotype (HLA-A*11-B*27-DRB1*04) occurred more frequently in patients with ESRD than in controls. In Brazil, Ravazzi-Gauch et al [15] reported that the most frequent alleles at the HLA-A, HLA-B, and HLA-DR loci were HLA-A*02, HLA-B*35, and HLA-DRB1*11, respectively. The most common haplotypes included A*01-B*08-DRB1*03 among White individuals and A*29-B*15-DRB1*04 among individuals of African ancestry.

Long and Sun [14] demonstrated that HLA-A*02, HLA-B*48, HLA-B*52, and HLA-B*55 may confer susceptibility to ESRD among the Han population in Hunan, China, whereas HLA-B60 appeared to be protective. Similarly, Iancu Loga et al [16] reported that HLA-A*11, HLA-A*34, HLA-A*69, HLA-B*41, HLA-B*50, HLA-DRB1*10, and HLA-DRB1*14 were positively associated with ESRD (OR >1). In contrast, HLA-DRB1*07, HLA-DRB1*08, and HLA-DRB1*13 demonstrated protective effects (OR <1). Furthermore, the 3-locus haplotype HLA-A*02-HLA-B*41-HLA-DRB1*03 exhibited a particularly strong association with ESRD (OR 3.15; *P*<.001).

The significance of HLA alleles extends beyond ESRD susceptibility, as they are also associated with the production of anti-HLA antibodies. The presence of anti-HLA antibodies poses a major challenge in kidney transplantation because they may lead to hyperacute or acute rejection [18,19]. Sensitization events capable of inducing anti-HLA antibody production include pregnancy, blood transfusion, and previous transplantation [20-24].

Interestingly, not all exposed individuals develop anti-HLA antibodies. Multiparous women and patients receiving repeated transfusions may remain unsensitized. Although exposure to HLA antigens through red blood cell transfusions increases the risk of anti-HLA antibody formation both in patients who are prekidney transplant and in patients who are postkidney transplant, substantial interindividual variability in immune responsiveness exists. This observation suggests that additional factors influence the immunological response leading to anti-HLA antibody production. One such factor may be the recipient’s HLA allele profile [20-22].

Recipient HLA patterns influence antigen presentation and T-cell activation. Recipient HLA molecules play a critical role in donor antigen recognition through the indirect recognition pathway. Recipient CD4^+^ T lymphocytes recognize donor-derived peptides presented by recipient antigen-presenting cells in association with HLA class II molecules. Peptide binding is determined by amino acid variations within the peptide-binding groove (hypervariable region) of HLA class II molecules. Consequently, recipient HLA alleles influence both the repertoire of donor peptides that can be presented and the magnitude of T-cell reactivity [25-27]. Therefore, our study aims to analyze the role of HLA allele patterns in the incidence of ESRD and anti-HLA antibody production. Moreover, rather than prespecifying a limited set of candidate alleles, the present study evaluates HLA-A, -B, -C, -DPA1, -DPB1, -DQA1, -DQB1, and -DRB1 to comprehensively characterize population-specific HLA allele and haplotype patterns associated with ESRD and anti-HLA antibody production.

## Methods

### Study Design and Setting

This is an observational study with a case-control design that incorporates both retrospective and prospective data collection. Retrospective HLA typing and HLA antibody data were obtained from medical records of patients with ESRD, including candidates for kidney transplant and patients undergoing routine hemodialysis, as well as healthy controls, including kidney donors and other clinically healthy individuals, between January 2020 and May 2026. Additional participants for both the case and control groups are being prospectively recruited between June and December 2026 according to the predefined inclusion and exclusion criteria.

The inclusion criteria for the patient group are participants who (1) are aged 18 years or older, (2) have been diagnosed with ESRD, (3) have complete HLA typing data for 8 loci, and (4) have HLA antibody data. The exclusion criterion for the patient group is patients who have undergone kidney transplantation. The inclusion criteria for the control group are patients who are aged 18 years or older and who are willing to participate in this study. The exclusion criterion for the control group is patients with impaired kidney function, defined as an eGFR of less than 90 mL/min/1.73 m².

Based on a pilot study on HLA typing of patients with ESRD who were scheduled to undergo kidney transplantation and their corresponding donors at Dr Sardjito General Hospital, Yogyakarta, several relatively predominant HLA alleles were identified as being potentially associated with ESRD, although the associations did not reach statistical significance. These alleles included HLA-B*15 (OR 1.46, 95% CI 0.94‐2.34), HLA-C*08 (OR 1.61, 95% CI 0.98‐2.65), HLA-DQB1*03 (OR 1.4, 95% CI 0.97‐2.06), and HLA-DRB1*12 (OR 1.17, 95% CI 0.79‐1.73). However, rather than limiting the analysis to these preliminary findings, the present study will comprehensively evaluate HLA allele and haplotype patterns across the investigated loci in relation to ESRD and HLA antibody production.

### Sample Size

The sampling method used in this study is consecutive sampling based on the order of arrival of patients with ESRD undergoing hemodialysis at the Integrated Renal Unit of Dr Sardjito General Hospital, Yogyakarta. This study will compare HLA allele patterns associated with ESRD; therefore, both case and control groups will be required.

Based on the study by Susianti et al [10], the proportion of cases carrying a specific HLA pattern, namely, a 3-locus haplotype, was 5.98% (P1), whereas the proportion among controls was 1.01% (P2), with an OR of 5.72 [10]. Assuming a 2-sided significance level (α) of .05 and a statistical power of 80% (*β*=.20), the minimum required sample size is calculated to be 150 participants in each of the case and control groups. The sample size was calculated using the case-control sample size formula available from OpenEpi [28].

### Data Collection and Analysis

Data will be collected by compiling HLA typing and HLA antibody data from patients with ESRD who are candidates for kidney transplantation, as well as HLA typing data from kidney transplant donors or healthy controls. Patient and donor demographic and clinical characteristics will be obtained from their medical records.

HLA antibody data will be used for the calculated panel reactive antibody (cPRA) score using an online HLA antibody calculator [29]. The cPRA results will be classified as negative when the cPRA is less than or equal to 5% and positive when the cPRA is greater than 5%, according to the criteria described by Heise et al [30].

HLA typing of the HLA-A, HLA-B, HLA-C, HLA-DPA1, HLA-DPB1, HLA-DQA1, HLA-DQB1, and HLA-DRB1 loci will be performed with a LABScan 3D analyzer (One Lambda Inc) using the sequence-specific oligonucleotide probe method. HLA typing results will be analyzed using HLA Fusion software (One Lambda Inc), which identifies HLA alleles by comparing the observed reaction patterns with reference patterns derived from published HLA gene sequences [31].

Anti-HLA antibody testing will be performed using a Luminex bead-based assay (DiaSorin) with LABScreen reagents (One Lambda Inc). Serum IgG antibodies against HLA class I and/or class II antigens will be detected based on the specific binding of patient antibodies to HLA antigens immobilized on fluorescent microbeads. The resulting antigen-antibody complexes will subsequently be detected using a phycoerythrin-conjugated antihuman IgG secondary antibody. The fluorescence intensity is directly proportional to the amount of bound antibody and will be expressed as mean fluorescence intensity. Fluorescence signals will be acquired using the LABScan3D analyzer [31], which functions as a flow cytometric bead analyzer for multiplex immunoassays in immunology and transplantation laboratories, particularly for HLA antibody detection and HLA typing.

### Data Analysis Plan

The frequencies of HLA alleles, 2-locus haplotypes, and 3-locus haplotypes will be determined by direct counting and will be expressed as percentages. The associations between HLA alleles and ESRD will be estimated using ORs with 95% CIs. A 2-tailed *P* value of <.05 will be considered statistically significant. An OR greater than 1 will be interpreted as indicating increased susceptibility to ESRD, whereas an OR of less than 1 will be considered indicative of a protective effect against ESRD. Univariate and multivariable analyses will be performed to evaluate the effects of potential confounding variables on the development of ESRD and anti-HLA antibody production. Statistical analyses will be conducted using IBM SPSS Statistics (version 25; IBM Corp), OpenEpi software (version 3.0.1) [28], and R software (version 4.6.1; R Foundation for Statistical Computing).

### Ethical Considerations

The study protocol has been reviewed and approved by the Medical and Health Research Ethics Committee of the Faculty of Medicine, Public Health and Nursing, Universitas Gadjah Mada, Yogyakarta, Indonesia (approval reference number KE/FK/0750/EC/2026). Written informed consent will be obtained from all participants before enrollment. Participants will receive comprehensive information regarding the study objectives, procedures, potential risks, and benefits, as well as their right to participate voluntarily and withdraw from the study at any time without consequences.

## Results

Participant recruitment and data collection began after approval of ethical consideration. At the time of manuscript submission, 280 subjects have been recruited, including 131 in the case group and 149 in the control group. Recruitment and data collection are expected to be completed by December 2026. Data analysis is planned to be completed by February 2027, with dissemination of the study findings through scientific publication anticipated by mid-2027.

## Discussion

### Anticipated Findings

CKD and ESRD remain major public health challenges worldwide. The burden of CKD continues to increase, with a growing number of patients progressing to ESRD, which requires kidney replacement therapy, including dialysis or kidney transplantation. Indonesia is among the countries with a substantial CKD burden, with a steadily increasing number of patients requiring dialysis each year [3]. The progression from CKD to ESRD is a complex process initiated by nephron injury followed by chronic inflammation, immune activation, oxidative stress, and ultimately, tubulointerstitial fibrosis. Persistent nephron injury results in a progressive loss of functional nephron mass. This loss is initially compensated by adaptive hyperfiltration in the remaining functional nephrons. However, sustained hyperfiltration further exacerbates nephron damage by increasing intraglomerular pressure, which, over time, leads to podocyte injury, proteinuria, and glomerulosclerosis [8,32].

The primary causes of kidney tissue injury are immunological mechanisms (mediated by either immune complexes or immune cells), tissue hypoxia, ischemia, endogenous substances such as glucose and paraproteins, and genetic factors. Tubulointerstitial injury may be further aggravated by proteinuria through several mechanisms, including direct tubular toxicity, alterations in tubular epithelial cell metabolism, increased cytokine synthesis, and upregulation of adhesion molecule expression. Tubular injury is further exacerbated when protein reabsorption exceeds the lysosomal degradative capacity of proximal tubular epithelial cells, resulting in lysosomal rupture and subsequent cellular damage [33,34].

HLA molecules are glycoproteins that present endogenous and exogenous antigens to T lymphocytes, enabling recognition by T-cell receptors and initiating antigen-specific immune responses [35]. The HLA region is among the most polymorphic regions of the human genome. Polymorphic variations in HLA genes give rise to amino acid sequence diversity within HLA molecules, thereby altering peptide-binding specificity. Consequently, HLA molecules encoded by different alleles present distinct repertoires of antigenic peptides to T lymphocytes [36]. This remarkable genetic diversity contributes to interindividual and interpopulation differences in disease susceptibility while conferring an evolutionary advantage through enhanced adaptability to diverse pathogens [37].

HLA polymorphisms influence antigen presentation by altering peptide-binding affinity as well as central and peripheral immune tolerance. These variations may modify the threshold for antigen recognition, thereby predisposing individuals to the loss of immune tolerance toward tissue-specific self-antigens, including renal antigens. Certain HLA alleles exhibit a high affinity for presenting self-peptides or pathogen-derived peptides that share structural homology with self-antigens, leading to aberrant T-cell activation through molecular mimicry. Together, these mechanisms underscore the pivotal role of HLA polymorphisms in genetic susceptibility to autoimmune responses and the development of chronic renal inflammation [8,38,39].

The association between HLA polymorphisms and ESRD is attributed to abnormal immune responses against self- and non–self-antigens, leading to persistent inflammation and a reduced capacity of the kidney to adapt to chronic injury. In autoimmune disorders such as systemic lupus erythematosus, HLA class II molecules present renal self-antigens to CD4^+^ helper T cells, leading to autoreactive immune responses, autoantibody production, and subsequent glomerular injury. These observations support the concept that HLA-mediated genetic susceptibility contributes to the initiation and progression of kidney disease through dysregulated immune mechanisms [40].

HLA may contribute to kidney disease through its immunological effects in both lymphoid organs and renal tissue. These effects are mediated by HLA-dependent activation of naive CD4^+^ and CD8^+^ T cells within secondary lymphoid organs. Antigen-specific CD4^+^ T cells are essential for humoral immune responses because they recognize antigenic peptides presented by HLA class II molecules on B cells, thereby inducing B-cell activation and differentiation into antibody-producing plasma cells. Furthermore, effector CD8^+^ T cells, along with T helper 1 (Th1) and T helper 17 (Th17) cells, contribute to renal injury by recognizing intrarenal antigens, including those presented by glomerular microvascular antigen-presenting cells, resulting in sustained renal inflammation and tissue damage [41].

The association between HLA and ESRD may be influenced by the relationship between specific HLA alleles and underlying diseases, including diabetes mellitus, glomerulonephritis, hypertension, and autoimmune disorders. One well-established example is diabetic kidney disease, which has been associated with the HLA-DRB1*04 and HLA-DQB1*03:02 alleles. A phenome-wide association study identified HLA-DQB1*03:02 as being associated with both kidney transplantation (OR 1.4) and an increased risk of type 1 diabetes mellitus and diabetic kidney disease (OR 7.1), suggesting that this allele contributes to disease susceptibility through its association with diabetes [42]. Genome-wide studies have also consistently demonstrated strong associations between HLA loci and glomerulonephritis, further supporting an indirect link between HLA polymorphisms and ESRD, particularly in populations where glomerulonephritis is a leading cause of kidney failure, such as in China. In a genome-wide association study involving 1194 Chinese Han patients with IgA nephropathy and 902 healthy controls, Gharavi et al [43] identified 3 independent susceptibility loci within the major histocompatibility complex, in addition to a common deletion of the *CFHR1* and *CFHR3* genes on chromosome 1q32 and another susceptibility locus on chromosome 22q12. All identified loci exceeded the threshold for genome-wide significance (with *P* values from the association analyses ranging from 1.59×10⁻²⁶ to 4.84×10⁻⁹), with minor allele ORs ranging from 0.63 to 0.80. Because genome-wide association studies simultaneously evaluate hundreds of thousands to millions of single-nucleotide polymorphisms, a stringent significance threshold of *P*<5 × 10⁻⁸ is conventionally applied to define genome-wide significance [43].

The influence of HLA on the susceptibility to and progression of ESRD is not always dependent on underlying kidney disease. Several studies have demonstrated direct associations between specific HLA alleles and the risk of ESRD independent of disease etiology. In a Taiwanese population, Dai et al [12] reported that HLA-DR3 (OR 1.9, 95% CI=1.10‐3.32; *P*=.024) and HLA-DR11 (95% CI=1.13‐3.76; *P*=.021) were associated with an increased risk of ESRD, whereas HLA-DR8 exhibited a protective effect (OR 0.47, 95% CI=0.24‐0.92; *P*=.027). In contrast, no significant associations were observed between the HLA-A or HLA-B alleles and ESRD susceptibility. Similarly, Cao et al [13] identified 5 HLA alleles (HLA-A*24, HLA-B*55, HLA-B*54, HLA-B*40(60), and HLA-DRB1*04) and 1 haplotype (HLA-A*11-B*27-DRB1*04) that occurred significantly more frequently in patients with ESRD than in healthy controls. In a Brazilian cohort, Ravazzi-Gauch et al [15] reported that the most prevalent alleles at the HLA-A, HLA-B, and HLA-DR loci were HLA-A*02, HLA-B*35, and HLA-DRB1*11, respectively. The most common haplotypes were HLA-A01-B08-DRB103 among individuals of European ancestry and HLA-A*29-B*15-DRB1*04 among individuals of African ancestry. In contrast, Crispim et al [44] reported different findings in a population from southern Brazil, with HLA-A*74 and HLA-DRB1*11 reported to be positively associated with ESRD. However, no HLA haplotypes were found to be significantly associated with ESRD susceptibility in this population.

HLA polymorphisms affect antigen presentation and CD4^+^ T-cell activation, which subsequently promote B-cell differentiation into plasma cells that produce anti-HLA antibodies. Recipient HLA molecules play a central role in donor antigen recognition through the indirect allorecognition pathway. In this pathway, recipient CD4^+^ T cells recognize donor-derived peptides presented by recipient antigen-presenting cells in association with HLA class II molecules. Peptide binding is determined by amino acid polymorphisms within the peptide-binding groove (hypervariable region) of HLA class II molecules. Consequently, the recipient HLA allele repertoire influences both the range of donor-derived peptides that can be presented and the magnitude of the ensuing T-cell response [25-27].

Anti-HLA antibody production is further influenced by previous sensitizing events, including blood transfusion, pregnancy, prior organ transplantation, and autoimmune diseases. Pregnancy represents a potent source of HLA sensitization because persistent fetal microchimerism exposes the maternal immune system to paternal HLA antigens, promoting helper T-cell activation, B-cell differentiation, and long-lasting anti-HLA antibody production [45]. Similarly, blood transfusion, particularly nonleukoreduced blood products, exposes recipients to donor leukocyte HLA antigens, thereby inducing both class I and class II anti-HLA antibodies. Repeated transfusions and the inflammatory milieu associated with CKD may further enhance antigen presentation and immune sensitization [46-48]. Autoimmune diseases also predispose individuals to anti-HLA antibody formation through dysregulated adaptive immune responses characterized by defective immune tolerance, chronic inflammation, and enhanced B-cell activation. In addition, genetic variants, including specific HLA class II alleles and immune regulatory genes, may augment antigen presentation and humoral immune responses, thereby increasing susceptibility to HLA sensitization [36,38,44]. Collectively, these sensitizing events contribute to the development of anti-HLA antibodies and may adversely affect subsequent kidney transplantation outcomes.

### Strength and Limitations

A notable strength of this study lies in its investigation of the dual role of HLA allele patterns as determinants of both susceptibility to ESRD and anti-HLA antibody production within the Indonesian population. Although previous studies conducted in different countries have demonstrated associations between specific HLA alleles and ESRD risk, the reported findings have been inconsistent, likely reflecting differences in ethnic background and environmental exposure that influence HLA allele distribution and their immunogenetic effects. Likewise, the relationship between HLA allele patterns and anti-HLA antibody production among patients with ESRD has been investigated in a limited number of studies, with considerable heterogeneity in both study findings and the types of anti-HLA antibodies evaluated. To date, no comparable study has been conducted in Indonesia despite the country’s extensive ethnic diversity and unique genetic background, which may substantially influence HLA allele frequencies and their associations with disease susceptibility and alloimmune responses.

Although this study is expected to provide important insights into the association between HLA alleles, ESRD, and anti-HLA antibody production in the Indonesian population, several limitations should be acknowledged. First, the case-control design allows identification of statistical associations but cannot establish temporal or causal relationships between specific HLA alleles and the development of ESRD or HLA sensitization. Second, because participants will be recruited from a single tertiary referral center, the study population may not fully represent the genetic diversity of the Indonesian population, thereby limiting the generalizability of the findings to other ethnic groups and health care settings. Third, the highly polymorphic nature of the HLA system results in numerous low-frequency alleles, which may reduce statistical power to detect significant associations for rare variants despite an adequate overall sample size. Finally, although major demographic and clinical variables will be collected, residual confounding from environmental exposures, primary kidney disease, dialysis duration, blood transfusion history, pregnancy, previous transplantation, autoimmune diseases, and other sensitizing events may not be completely eliminated and could influence both HLA antibody production and disease susceptibility. Prospective multicenter studies with larger ethnically diverse populations, high-resolution HLA genotyping, and longitudinal immunological follow-up are warranted to validate and extend the findings in this study.

### Conclusions

This study aims to evaluate the role of HLA class I and class II allele patterns in the development of ESRD and anti-HLA antibody production. Furthermore, the effects of demographic characteristics, sensitization-related factors (including blood transfusion, pregnancy, and autoimmune disease), and clinical comorbidities on HLA antibody production and ESRD susceptibility will also be investigated. Therefore, this study is expected to provide the first comprehensive evaluation of HLA alleles associated with ESRD risk and anti-HLA antibody production while also characterizing the spectrum of anti-HLA antibodies and the factors influencing their development in the Indonesian population. These findings may contribute to population-specific immunogenetic evidence and provide a foundation for future risk stratification and personalized approaches in renal transplantation.

## Abbreviations

CKD: chronic kidney disease
cPRA: calculated panel reactive antibody
eGFR: estimated glomerular filtration rate
ESRD: end-stage renal disease
HLA: human leukocyte antigen
or: odds ratio
